# ROCK1 Induces Endothelial-to-Mesenchymal Transition in Glomeruli to Aggravate Albuminuria in Diabetic Nephropathy

**DOI:** 10.1038/srep20304

**Published:** 2016-02-04

**Authors:** Hui Peng, Yuanqing Li, Cheng Wang, Jun Zhang, Yanru Chen, Wenfang Chen, Jin Cao, Yanlin Wang, Zhaoyong Hu, Tanqi Lou

**Affiliations:** 1 Department of Internal Medicine, Nephrology Division, the Third Affiliated Hospital of Sun Yat-sen University, Guangzhou, 510630, China; 2 Department of Medicine, Nephrology Division, Baylor College of Medicine, Houston, Texas, 77030-3411, U.S; 3 Department of Pathology, the First Affiliated Hospital of Sun Yat-sen University, Guangzhou, 510080, China

## Abstract

Endothelial-to-mesenchymal transition (EndMT) can cause loss of tight junctions, which in glomeruli are associated with albuminuria. Here we evaluated the role of EndMT in the development of albuminuria in diabetic nephropathy (DN). We demonstrated that EndMT occurs in the glomerular endothelium of patients with DN, showing by a decrease in CD31 but an increase in α-SMA expression. In glomeruli of *db*/*db* mice, there was an increased ROCK1 expression in the endothelium plus a decreased CD31-positive cells. Cultured glomerular endothelial cells (GEnCs) underwent EndMT when stimulated by 30 mM glucose, and exhibited increased permeability. Meanwhile, they showed a higher ROCK1 expression and activation. Notably, inhibition of ROCK1 largely blocked EndMT and the increase in endothelial permeability under this high-glucose condition. In contrast, overexpression of ROCK1 induced these changes. Consistent alterations were observed *in vivo* that treating *db*/*db* mice with the ROCK1 inhibitor, fasudil, substantially suppressed the expression of α-SMA in the glomerular endothelium, and reduced albuminuria. Thus we conclude that ROCK1 is induced by high glucose and it stimulates EndMT, resulting in increased endothelial permeability. Inhibition of ROCK1 could be a therapeutic strategy for preventing glomerular endothelial dysfunction and albuminuria in developing DN.

Diabetic nephropathy (DN), a leading cause of end-stage renal disease, is classed as a microvascular complication of diabetes^1^. Early characteristic manifestations of DN are glomerular hyperfiltration and the development of microalbuminuria. A potential pathogenetic mechanism of DN is that glomerular endothelial cells (GEnCs) are injured resulting in increased filtration of albumin^2^. In response to injury, endothelial cells are converted into mesenchyme-like cells, a process called endothelial-to-mesenchymal transition (EndMT)^3^. In fact, EndMT results in loss of endothelial characteristics and increased characteristics of mesenchymal cells. EndMT is also important because it adversely changes the plasticity and integrity of endothelial cells to contribute to the pathogenesis of many diseases^4^,^5^,^6^. Hyperglycemia could initiate pathological processes in endothelial cells by stimulating EndMT^1^,^7^,^8^. EndMT also reportedly contributes to the development of interstitial fibrosis and vascular angiogenesis in DN and other kidney diseases^8^,^9^. How EndMT is activated to affect the pathogenesis of GEnCs in DN is unclear.

Rho-associated kinase 1 (ROCK1) is a protein serine/threonine kinase. It acts to regulate the actomyosin cytoskeleton and participates in biological and pathological processes such as cell polarity, cell motility, tumor metastasis and epithelial-to -mesenchymal transition (EMT)^10^. ROCK1 can affect the pathogenesis of DN: Wang *et al.* reported that activation of ROCK1 results in dysfunction of GEnCs leading to albuminuria in mouse models of DN^11^. We find that ROCK1 is involved in hyperglycemia-induced permeability of cultured GEnCs in early stages of DN. The mechanism depends on RhoA activation^12^. Other components of the mechanism by which ROCK1 activation stimulates glomerular endothelial hyperpermeability are incompletely understood.

Apart from affecting endothelial cells, ROCK1 activation has been linked to EMT^13^,^14^. In that report, epithelial cells underwent conversion to mesenchymal cells. Besides activating ROCK1, TGF-β-mediated Rho signals can contribute to the development of both EMT and EndMT^15^. Given these experiments, we hypothesized that ROCK1 activation stimulates EndMT in GEnCs, leading to altered integrity of GEnCs and resulting in albuminuria. We examined whether EndMT is present in glomeruli of patients with DN and whether a high concentration of glucose will cause EndMT in GEnCs *in vitro* and *in vivo*. Finally, we examined whether ROCK1 stimulated by 30 mM glucose will trigger EndMT.

## Results

### Evidence for EndMT in GEnCs of patients with DN and *db*/*db* diabetic mice

**Figure 1:**
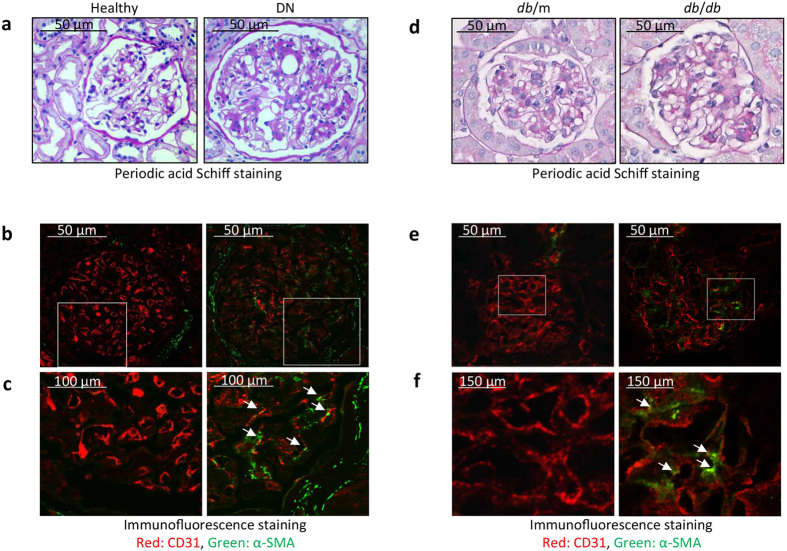
Evidence for EndMT in glomeruli of patients with DN and *db/db* diabetic mice. (**a)** Periodic acid Schiff staining of healthy kidney section (left) and the kidney section from patient with DN (right). (**b**) Confocal microscopy reveals that mesenchymal marker (α-SMA, green) co-localized with endothelial marker (CD31, red) in glomerulus of patient with DN. (**c**) Corresponding higher magnification of selected area in (**b)**. White arrows indicate that CD31-positive cells express α-SMA. (**d**) Periodic cid Schiff staining of the glomerulus of *db*/m (left) and *db/db* mice with early stage of DN (right). (**e**) Confocal microscopy reveals that α-SMA and CD31 were co-stained in glomerulus of *db/db* mice with early stage of DN (right). Double immunofluorescent staining of CD31 (red) and α-SMA (green). (**f**) Corresponding higher magnification of selected area in (**e)**. White arrows indicate the co-localization of CD31 and α-SMA.

To evaluate whether EndMT occurs, we examined renal biopsy sections of patients who were diagnosed with type 2 diabetes and DN. Expressions of CD31 and α-SMA in glomeruli were examined. As shown in Fig. 1, glomeruli of a patient with DN exhibited glomerular hypertrophy, mesangial proliferation, and glomerular basement membrane thickening (Fig. 1a). Double immunofluorescence staining revealed that, CD31 was reduced in the glomerular endothelium of patients with DN compared to results found in healthy individuals (Fig. 1b,c, red). Importantly, the decrease in the endothelial marker, CD31, in diabetic kidneys associated with an increase in the mesenchymal marker, α-SMA, in the glomerular endothelium (Fig. 1b,c, green). These results suggest that EndMT might have occurred in the glomerular endothelium of patients with DN. Similar changes were detected in glomeruli of *db*/*db* diabetic mice (Fig. 1d–f).

### High glucose induces EndMT in cultured GEnCs

**Figure 2:**
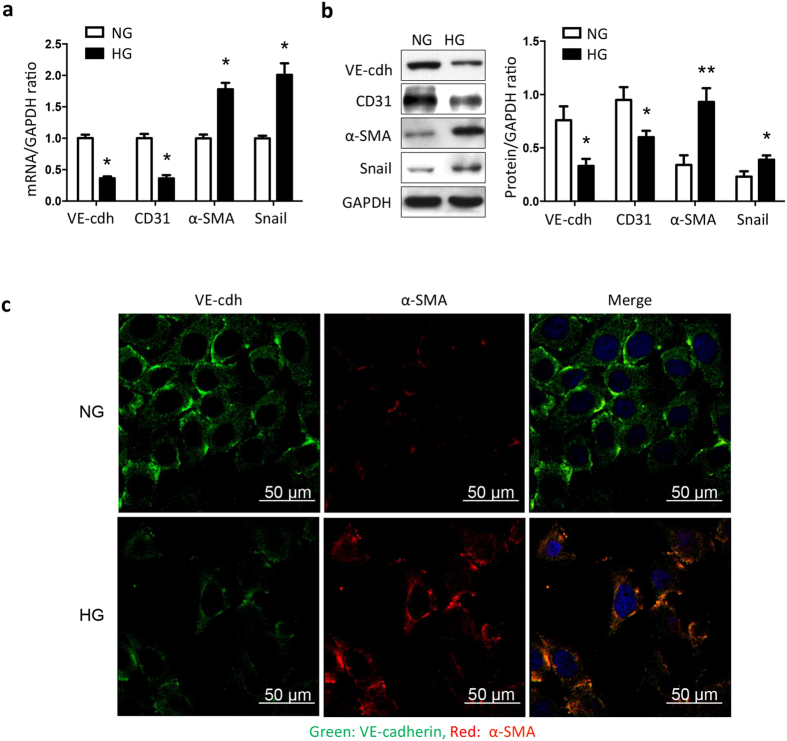
High glucose induces EndMT in cultured GEnCs. GEnCs were treated with 5.5 mM glucose (normal glucose, NG) or 30 mM glucose (high glucose, HG) for 5 days. (**a**) mRNA levels of endothelial markers (VE-cadherin, CD31) and mesenchymal markers (α-SMA, Snail) were accessed using real-time PCR. (**b**) protein levels of VE-cadherin, CD31, α-SMA and Snail were accessed with immunoblotting. (**c**) Immunofluorescence double-staining of VE-cadherin (green) and α-SMA (red) in GEnCs treated with NG and HG. Values are mean ± SEM from 5 experiments, *P < 0.05 vs. NG (t-test). **P < 0.01 vs. NG (t-test). NG: normal glucose, HG: high glucose, VE-cdh: VE-cadherin.

Since hyperglycemia is a characteristic of diabetes and is linked to endothelial damage^16^,^17^, we investigated whether adding 30 mM glucose to GEnCs would stimulate EndMT. After 5 days of exposure to 30 mM D-glucose, GEnCs exhibited reduced mRNA levels of endothelial markers (CD31, VE-cadherin) but an increase in markers of mesenchymal cells (α-SMA, Snail) (Fig. 2a). Similarly, immunoblotting demonstrated that the expressions of endothelial markers, CD31 and VE-cadherin, were significantly reduced by 30 mM glucose (*P* = 0.0279 and 0.0165, by t-test respectively), while the expressions of EndMT markers, α-SMA and Snail changed reciprocally (Fig. 2b). Results of immunofluorescence staining were consistent with these observations. In GEnCs, 30 mM glucose induced reciprocal changes in expressions of endothelial markers (VE-cadherin) and mesenchymal markers (α-SMA) (Fig. 2c). Notably, there also were morphological changes of GEnCs: in response to 5.5 mM glucose, the cells were cobble-stone shaped and tightly interconnected, but in response to 30 mM glucose, the cells tended to elongate and lose their cell-to-cell connections (Fig. 2c). Collectively, these results demonstrate that high glucose can stimulate EndMT in GEnCs. We examined whether our results were confounded by osmotic events. In each experiment, we evaluated responses to both 30 mM glucose and 24.5 mM mannitol plus 5.5 mM glucose. In each case, responses to the mannitol/glucose mixture were not statistically different from responses occurring in cells incubated with 5.5 mM glucose alone (see Supplementary Fig. S1).

### ROCK1 activation in GEnCs by high glucose is associated with EndMT

**Figure 3:**
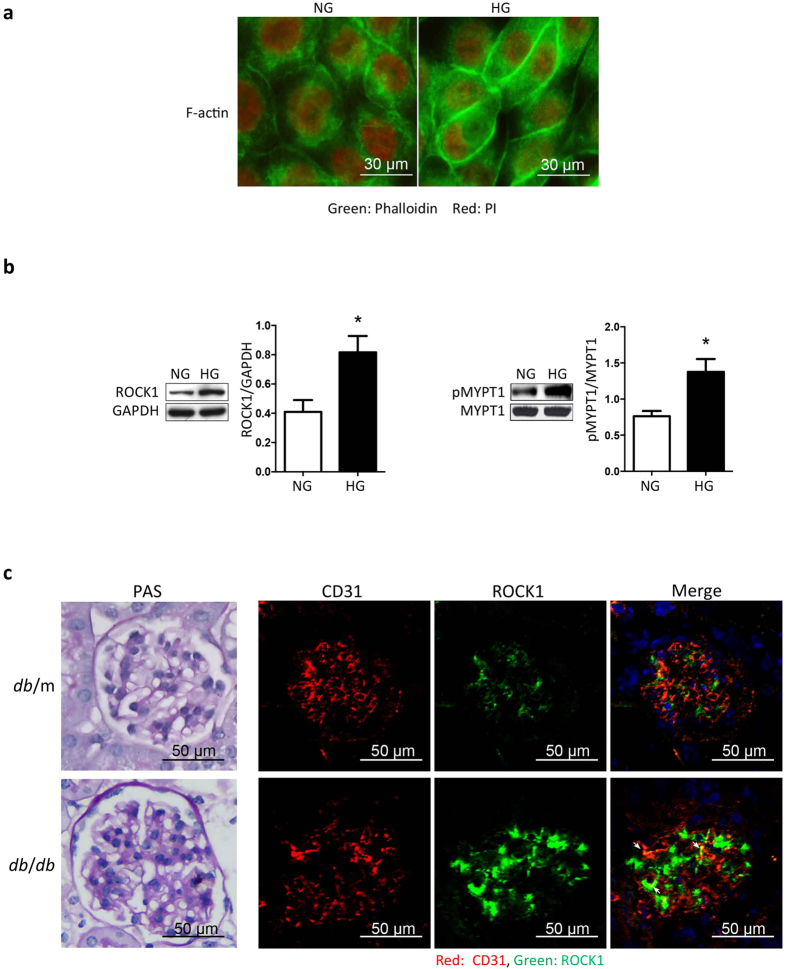
High glucose mediates ROCK1 activity in glomerular endothelial cells. (**a)** GEnCs were treated with NG or HG for 48 hr, the F-actin was visualized with Phalloidin - Fluor 488 (green), nuclei were stained with Propidium iodide (PI, Red). (**b**) GEnCs were treated with NG or HG. ROCK1 expression and activity were detected by immunoblotting. ROCK1 activity was assessed by the level of phosphorylated MYPT1 (p-MYPT1). Values are mean ± SEM from 5 experiments. *P < 0.05 vs. NG (t-test). NG: normal glucose, HG: high glucose. (**c**) Paraffin sections (left panel) of kidneys of *db*/m and *db/db* mice were stained with Periodic acid-Schiff (PAS). Cryosections (right panel) of kidneys of *db*/m and *db/db* mice were stained with CD31 (red) and ROCK1 (green). CD31 and ROCK1 colocalizations are indicated by arrows.

Since cell shape is largely depended on cytoskeleton organization, we therefore examined cytoskeleton remodeling in GEnCs treated with normal or high glucose. With phalloidin staining, we found that the actin filaments (F-actin) extended across the entire cell with normal glucose, while high glucose caused rearrangement of actin filaments leading to their transit to the periphery of the cell (Fig. 3a). Because cytoskeleton rearrangement was governed by RhoA GTPase and its downstream ROCK1 signaling, this result suggested that high glucose might activate ROCK1 in GEnCs. Indeed, immunoblotting showed that ROCK1 expression in cultured GEnCs was significantly increased (*P* = 0.0186, t-test. Fig. 3b, left panel). ROCK1 activity, evaluated from assessing the level of phosphorylated MYPT (p-MYPT), also increased in response to high glucose (Fig. 3b, right panel). To ascertain if there is a linkage between ROCK1 and EndMT, we assessed ROCK1 expression and activity in the glomeruli of 16-week old *db*/m control and diabetic *db*/*db* mice with DN (Fig. 3c, left panel). Double-immunostaining revealed that ROCK1 co-localized with CD31 in control mice (*db*/m), whereas in *db*/*db* mice, ROCK1 expression markedly increased in both CD31 positive cells and in mesangial cells (Fig. 3c, right panel). These results suggest that ROCK1 activation could be involved in mediating EndMT that is induced by high glucose in glomerular endothelium.

### ROCK1 mediates high-glucose-induced EndMT in cultured GEnCs

**Figure 4:**
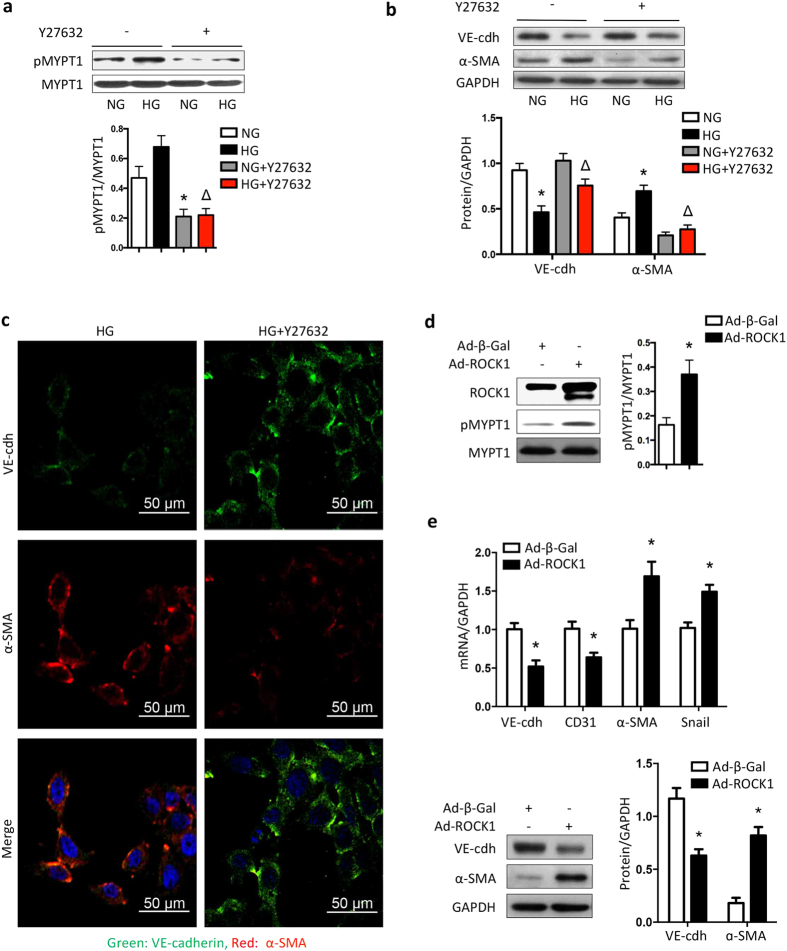
ROCK1 mediates high-glucose-induced EndMT in cultured GEnCs. (**a)** GEnCs were treated with HG in the presence or absence of ROCK inhibitor Y27632 (10 μmol/L), ROCK1 activity was assessed with immunoblotting using anti-pMYPT antibody. (**b**) VE-cadherin and α-SMA expressions were accessed using immunoblotting. Values are mean ± SEM from 5 experiments. *P < 0.05, vs. NG , Δ*P* < 0.05, vs. HG (two-way ANOVA, followed by Bonferroni’s multiple comparisons test). (**c**) Morphological characteristics were observed using immunofluorescent microscopy (400×). Red and green represented α-SMA and VE-cadherin respectively. (**d)** GEnCs were infected with adenovirus containing ROCK1-overexpression cassette for 2 days under NG. The ROCK1 expression and activity were examined with immunoblotting. Values are mean ± SEM from 3 experiments. *P = 0.03 vs. Ad-β-Gal (t-test). (**e**) mRNAs and proteins of VE-cadherin and α-SMA were determined using real-time PCR (upper panel) and immunoblotting (lower panel). Values are mean ± SEM from 3 experiments. *P < 0.05 vs. Ad-β-Gal (t-test). NG: normal glucose, HG: high glucose, VE-cdh: VE-cadherin; Ad-β-Gal: adenovirus containing β-galactosidase cassette; Ad-ROCK1: adenovirus containing ROCK1-overexpression cassette.

To examine if ROCK1 activation is necessary for glucose-induced EndMT in GEnCs, we treated GEnCs with a ROCK1 inhibitor, Y27632, before adding 30 mM glucose (Fig. 4a). As shown in Fig. 4b, inhibition of ROCK1 substantially blocked the reduction in VE-cadherin in comparison to results of cells not exposed to the inhibitor. As expected, ROCK1 inhibition blunted the increase in α-SMA in GEnCs incubated with 30 mM glucose (Fig. 4b). These results were confirmed by immunofluorescence staining (Fig. 4c). Next, we used an adenoviral approach to overexpress ROCK1 in GEnCs. Forced expression of ROCK1 resulted in a significant ROCK1 activation (Fig. 4d). The increase in ROCK1 activity suppressed VE-cadherin while increased α-SMA at both transcriptional and translational levels (Fig. 4e). These results indicate that ROCK1 activation induced by high glucose can mediate EndMT in GEnCs.

### ROCK1 inhibition prevents high-glucose-induced hyperpermeability in GEnCs

**Figure 5:**
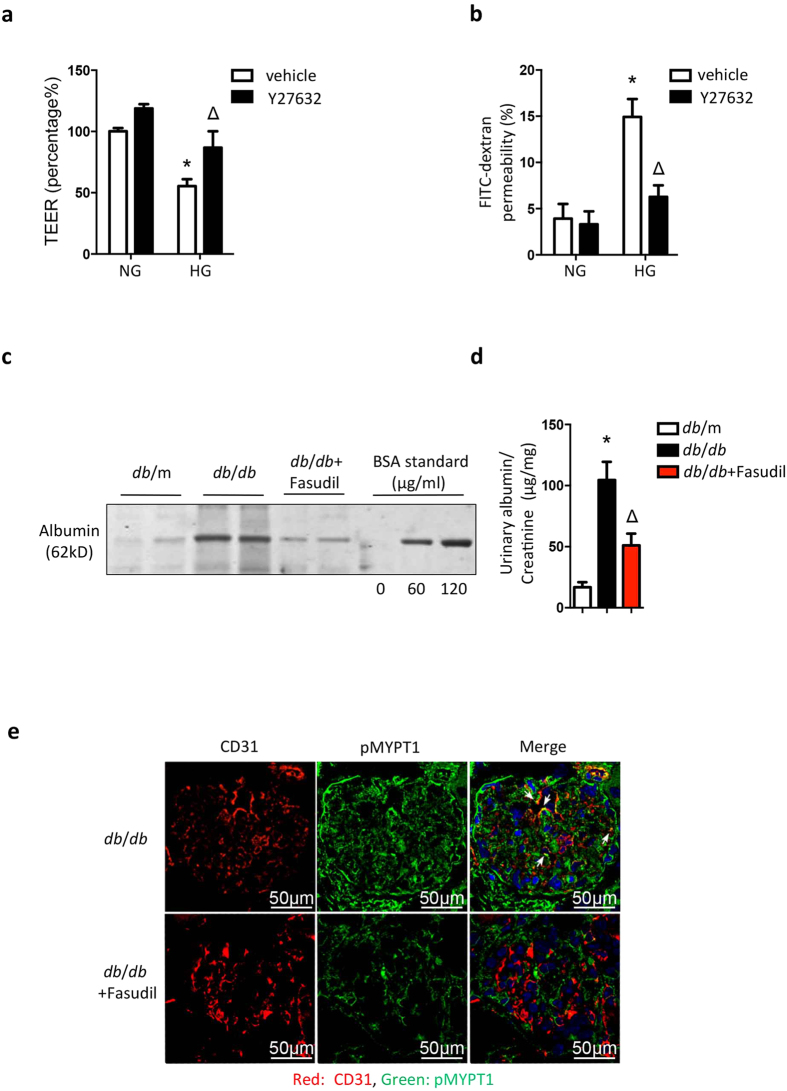
ROCK1 inhibition prevents high-glucose-induced hyperpermeability in GEnCs and proteinuria in mice with early stage of DN. (**a**) 70% confluent GEnCs plated on transwell inserts were treated with 30 mM glucose for 5 days with or without ROCK1 inhibitor, Y27632 (10 μmol/L). Endothelial barrier function was determined by measuring trans-endothelial electrical resistance (TEER). (**b**) With same treatment as in (**a**) the permeability of GEnCs was examined by the amount of FITC-dextran across GEnCs monolayer. Values are mean ± SEM from 3 experiments. *P < 0.01, vs. NG, Δ*P* < 0.01, vs. HG (two-way ANOVA, followed by Bonferroni’s multiple comparisons test). (**c**) 12-week-old *db*/m or *db/db* mice were treated with saline or fasudil (ROCK1 inhibitor, 10 mg/kg/day, intraperitoneal injection, i.p.) for 4 weeks. 24-h urine was collected and applied for SDS-PAGE followed by Coomassie brilliant blue staining. (**d**) The albumin excretion was accessed with urinary albumin/creatinine ratio. Values are mean ± SEM from 5 mice in each group. *P = 0.0012 vs. *db*/m. Δ*P* = 0.0231, vs. *db/db* (two-way ANOVA, followed by Bonferroni’s multiple comparisons test). NG: normal glucose, HG: high glucose. (**e**) Cryosections of kidneys (from mice described in (**c**) were subjected to double immunofluorescent staining with CD31 (red) and p-MYPT1 (green). Arrows indicate p-MYPT1 staining in CD31-positive cells.

Since 30 mM glucose induces EndMT and leads to increased permeability, we examined whether ROCK1 inhibition would preserve the integrity of cultured GEnCs. We examined how the inhibition of ROCK1 altered glucose-induced changes in barrier function in GEnCs exposed to 30 mM glucose. As shown in Fig. 5a, incubation with 30 mM glucose sharply decreased trans-endothelial electrical resistance (TEER) in GEnCs to 55.4%, but in cells pretreated with the ROCK1 inhibitor, Y27632, TEER was restored to 86.8%. Similarly, glucose-induced hyperpermeability in GEnCs was attenuated by the ROCK1 inhibitor (Fig. 5b). Next, we examined if inhibition of ROCK1 would suppress albuminuria in *db*/*db* mice in an early stage of DN. We treated 12-week-old *db*/*db* mice with fasudil, a ROCK inhibitor, for 4 weeks and marked suppression of albuminuria was uncovered, as shown in Fig. 5c,d. Fasudil also suppressed ROCK1 activity (evaluated from immunostaining of p-MYPT) in glomeruli (Fig. 5e). Based on these results, we conclude that ROCK1 inhibition can inhibit EndMT in GEnCs and hence, improve glomerular endothelial dysfunction even under a high-glucose circumstance.

## Discussion

DN is usually classified as a microvascular complication of diabetes, so we examined changes in glomerular endothelial cells which we know will affect the development and progression of micro-albuminuria in DN^1^. For example, decreased Klf2 expression in cultured GEnCs is associated with damage to the endothelial cells and podocytes of glomeruli during the early stage of DN^18^. Damage to endothelial cells and podocytes could be explained by defects in nitric oxide metabolism^19^,^20^. Indeed, it has been proposed that endothelial-cell-induced damage to podocytes is involved in increased oxidative stress in GEnCs and the development of focal segmental glomerular sclerosis (FSGS)^21^. These reports emphasize that dysfunction of GEnCs occurs during the pathogenesis of nephropathy. We described how activation of ROCK1 in GEnCs interferes with the expressions of tight junction proteins in the early stages of DN in our previous report, however, the mechanism that activates ROCK1 leading to the dysfunctions of glomerular endothelial cells was not identified^12^. In the current study, we find that EndMT occurs in the glomerular endothelium of patients and *db*/*db* mice with early stage of DN, as well as in cultured GEnCs. After documenting the presence of EndMT in GEnCs, we uncovered that activation of ROCK1 can be triggered by high concentrations of glucose and will mediate EndMT in GEnCs.

EndMT is considered as an extreme form of endothelial cell plasticity^22^,^23^. During EndMT, endothelial cells lose the specific markers, CD31 and VE-cadherin, but gain mesenchymal markers such as α-SMA, FSP1, Snail and SM22α. In the process of EndMT, the endothelial cells lose their attachment to each other, forming elongated mesenchymal cells^3^. These results were not only found in cultured GEnCs but were also present in the glomerular endothelium of patients with DN (Fig. 1a). We did not detect an increase in the expressions of typical EndMT markers, such as FSP1 and SM22α in cultured GEnCs or in glomeruli of *db*/*db* mice with early DN. Thus, phenotypic changes in GEnCs induced by high glucose *in vitro* and *in vivo* may reflect dedifferentiation identified as the regression of specialized cell to a simpler, more embryonic stage^24^. Thus, glomerular endothelial cells do not express their specific proteins. They undergo EndMT and exhibit a decrease in CD31 expression and increased permeability in endothelium. Neovascularization can occur in diabetic nephropathy both in glomeruli and interstitial, this process is associated with increased CD31 expression^25^,^26^. In diabetic nephropathy, the early changes in glomeruli are glomerular enlargement, followed by mesangial matrix expansion and thickened GBM. At this stage, neovascularization does not occur while later, there is loss or narrowing of capillaries and neovascularization may occur. In our studies, we focus on the early phase of diabetic nephropathy and hence, the results of CD31 staining did not reflect the neovascularization. Our observations are consistent with published results^27^,^28^.

ROCK1 is a multifunctional, serine/threonine kinase that regulates mitochondrial fission, apoptosis and cytoskeletal remodeling^11^,^29^,^30^. In endothelial cells, ROCK1 induces stress fiber rearrangement and appears to regulate the process of phenotypic changes in endothelial cells. For example, ROCK-1-null mice have reduced numbers of expression of CD34^+^/CD45^+^ fibrocytes that converts into myofibroblasts^31^. In our study, stimulating cells with high glucose, we observed the rearrangement of cytoskeleton indicating the activation of ROCK1. Additional, cytoskeleton rearrangement may result in morphological changes in GEnCs due to its connection with junctional molecules like VE-cadherin^32^. Earlier, we reported that activation of RhoA/ROCK1 in diabetic mice can damage the integrity of GEnCs by suppressing the expression of tight junctions’ proteins, ZO-1/occludin. In the current study, we show that ROCK1 activation stimulates the development of EndMT in GEnCs. Importantly, when ROCK1 was inhibited, the EndMT was abolished in cultured GEnC treated with high glucose. The dependence on ROCK1 was also demonstrated *in vivo* by using ROCK1 inhibitor, fasudil, which suppressed EndMT in the glomeruli of *db*/*db* mice with early stage of DN.

When endothelial cells undergo EndMT, cell-cell adhesion decreases and the permeability of the endothelium increases. ROCK1 activation is reported to affect cell-cell adhesion by regulating the interaction between actin filaments and intracellular junction molecules (tight junctions and adherence junctions)^10^. Our results support this conclusion because we found when ROCK1 was inhibited, the high glucose-induced morphological changes, namely “cobble-stone” appearance to a spindle-like shape, were blocked in GEnCs. ROCK1 inhibition also blunted α-SMA expression and an increase in endothelial permeability. Since TGF-β can activate ROCK1 via small GTPases^33^, ROCK1 could be a downstream effector of TGF-β.

We chose to study *db*/*db* mice on a C57BLKS/J background because albuminuria is detectable at 10 weeks even though there is no significant mesangial expansion or glomerulosclerosis. Notably, we detected α-SMA is expressing in CD31-positive glomerular cells in mice with microalbuminuria. We speculate that EndMT was already initiated in glomeruli at this stage of DN.

In conclusion, we find that EndMT is present in the kidneys of patients with DN. We demonstrated that EndMT is an important early change occurring in GEnCs of mice with DN or in cultured GEnCs that are exposed to a high-glucose media. Importantly, as glucose concentration increases, ROCK1 is activated to induce EndMT in GEnCs. This response the permeability of the endothelium and aggravates microalbuminuria. Inhibition of ROCK1 both prevents EndMT in GEnCs and the development of albuminuria, suggesting a potential therapeutic strategy to combat the development of DN.

## Materials and Methods

### Animal studies

All animal studies were approved and supervised by the Animal Care and Use Committee of Sun Yat-sen University, and were carried out in accordance with the approved guidelines. Controls (*db*/m) and diabetic mice (*db*/*db*, C57BLKS/J background) were purchased from the Model Animal Research Center of Nanjing University. 12-week-old mice received normal saline or fasudil (10 mg/kg/day, i.p.) for 4 weeks. Subsequently, 24-h urine samples were collected from mice in metabolic cages. Urine was subjected to SDS-PAGE and Coomassie brilliant blue staining. Urine creatinine was measured using the Creatinine Colorimetric Assay Kit (Cayman Chemical, Michigan, US).

### Cell culture and transfection

Rat GEnCs were kindly provided by Professor F. R. Danesh (MD Anderson cancer center, Houston, TX) and maintained at 37 °C in a humidified atmosphere containing 5% CO_2_, cells were cultured in RPMI 1640 Medium (Gibco by Invitrogen, Carlsbad, CA) containing 5.5 mM D-glucose (Sigma-Aldrich, St. Louis, MO), 10% fetal bovine serum (Gibco by Invitrogen), and 10% Nu-Serum I Culture Supplement (BD Biosciences, San Jose, CA). At 70% confluence, GEnCs were treated with or without 30 mM D-glucose in the presence or absence of a selective ROCK inhibitor, Y27632 (10 μM; Sigma-Aldrich). Mannitol (24.5 mM) was used as an osmotic control. To overexpress ROCK1, a ROCK1 adenovirus (Ad-ROCK1, Vectorbiolabs, Philadelphia, PA) was added to the medium bathing GEnCs at 1:2,000 dilutions for 2 days. β-galactosidase adenovirus (Ad-β-Gal, Vectorbiolabs, Philadelphia, PA) was used as a control for virus infection.

### Trans-endothelial electrical resistance assay

The TEER was used to evaluate the endothelial barrier function^34^ and was measured using the Millicell-Electrical Resistance System. Briefly, GEnCs were seeded onto Transwell inserts (0.4-μm pore size; Corning Costar, NY). At 70% confluence, initial TEER was measured, followed by treatment as described above. Final TEER was recorded when the treatment was completed. Electrical resistance was recorded from probes inserted into the buffer until similar values were obtained on three consecutive measurements. Changes in TEER value were calculated by subtracting initial resistance from final one and was shown as fold change.

### Permeability assay

The permeability of GEnCs was determined by measuring the diffusion of FITC-dextran (Relative molecular mass, 40 kDa; Sigma-Aldrich, MO) across a monolayer of endothelial cells with modifications. At the end of the experiment, the culture media in the upper chamber was replaced by the media containing 1 mg/mL FITC-dextran, and the FITC fluorescence (excitation, 492 nm; emission, 530 nm) was detected in the lower chamber 12 hours later using a SpectraMax M5 microplatereader (Molecular Devices, Sunnyvale, CA).

### Real-time PCR

Total RNA from cultured cells was isolated using RNAiso Plus (TAKARA, Shiga) and used in real-time PCR at a final concentration of 350 ng/10 μL. RT-PCR was conducted using an RT-PCR Reagent Kit (TAKARA, Shiga) and PCR Amplifier (Bio-Rad, Hercules, CA). Real-time PCR was conducted using SYBR Green PCR Reagents (Takara, Shiga) and a 7500 Real-time PCR System (Applied Biosystems, Invitrogen). Primers are shown as followed.

Rat α-SMA Forward: 5′-TGCCATGTATGTGGCTATTCA-3′

Reverse: 5′-ACCAGTTGTACGTCCAGAAGC-3′

Rat CD31 Forward: 5′-GTGGAAGTGTCCTCCCTTGA-3′

Reverse: 5′-GGACAGGGCTGGTTCATAAAT-3′

Rat Snail Forward: 5′-CTTGCGTCTGCACGACCT-3′

Reverse: 5′-CTTCACATCCGAGTGGGTCT-3′

Rat GAPDH Forward: 5′-GGCACAGTCAAGGCTGAGAATG-3′

Reverse: 5′-ATGGTGGTGAAGACGCCAGTA-3′

Rat VE-cadherin Forward: 5′-GAAGAAACCACTGATTGGCACTGTG-3′

Reverse: 5′-TTATACCAGGCGTGGGTTTCTCTGT-3′

Primer sequences for genes were analyzed by real-time PCR. Relative mRNA expressions were calculated using ΔΔCt analysis.

### Antibodies

Anti-VE-cadherin, CD31, and Snail antibodies were obtained from Santa Cruz Biotechnology (Santa Cruz, CA). Anti-α-SMA antibody was obtained from Sigma-Aldrich (St. Louis, MO). Anti-p-MYPT1and anti-MYPT1 antibodies were obtained from Millipore (Billerica, MA). An anti-ROCK1 antibody was obtained from Cell Signaling Technology (CST, Danvers, MA). Alexa Fluor 546 anti-rabbit IgG and Alexa Fluor 488 anti-mouse IgG antibodies were obtained from Invitrogen (Carlsbad, CA). An anti-GAPDH antibody was obtained from Proteintech Group (PTG, Chicago, IL). Peroxidase-conjugated anti-mouse and anti-rabbit secondary antibodies were obtained from Boster (Wuhan, Hubei).

### Immunoblotting

Cell proteins were prepared using a cell lysate solution (KeyGen Biotech, Nanjing), containing 10% proteinase inhibitor (Roche, Basel, Switzerland). Proteins were separated on 10% to 12% SDS-polyacrylamide gels and electrophoretically transferred onto polyvinylidene fluoride membranes. The membranes were incubated overnight at 4 °C with primary antibodies. The next day, the membranes were incubated with secondary antibodies at room temperature for 1 hour. Signals were detected by ECL Plus (Millipore) and captured by X-ray films (Fuji, Shizuoka). The films were scanned with a GS-800 Calibrated Densitometer (Bio-Rad), and the densitometry was analyzed using the Quantity One software.

### Histology and immunofluorescence

Studies of human samples were approved and supervised by the Ethics Committee of the Third Hospital of Sun Yat-sen University (Guangzhou, China). All protocols were conducted in accordance with the approved guidelines, and written informed consent was obtained from each subject. Normal kidney tissue (from adjacent healthy tissue of dissected kidney cancer samples) and renal-biopsy specimens (from type 2 diabetes patients with the diagnosis of diabetic nephropathy) were prepared from 3-μm frozen sections and fixed with acetone, periodic acid-Schiff (PAS) staining were conducted according to the manufacturer’s instructions. DN was diagnosed based on the presence: the history of type 2 diabetes, clinical syndrome characterized by persistent albuminuria (>300 mg/24 h), following diabetic lesions such as glomerulosclerosis, mesangial expansion, and diffuse thickening of the glomerular basement on electron microscopy. For immunofluorescence, sections were blocked by goat serum at room temperature and incubated with primary antibodies at 4 °C overnight. The next day, the sections were incubated with Alexa Fluor-labeled secondary antibodies at room temperature for 30 minutes. DAPI or Propidium iodide (PI) staining was used to visualize nuclei. GEnCs were grown on cover glasses and at the end of treatment, the cells were fixed by exposure to 4% paraformaldehyde for 10 min, incubated in 0.25% Triton X-100 for 7 minutes and blocked by goat serum at room temperature for 1 h. Incubation of primary and secondary antibodies was as described above for tissue staining. For detecting F-actin, cells were incubated with phalloidin labeled with Alexa Fluor 488 (Invitrogen, Carlsbad, CA) for 30 minutes at room temperature. Images were then obtained by the light microscopy or via the laser scanning confocal microscope (LSM 510 META; Carl Zeiss Microimaging, NY).

### Statistical analysis

Each experiment was repeated at least 3 times. Data are shown as means ± SEM and were evaluated using GraphPad Prism 6. All tests were two-tailed. Differences were considered significant if *P* < 0.05.

## Additional Information

**How to cite this article**: Peng, H. *et al.* ROCK1 Induces Endothelial-to-Mesenchymal Transition in Glomeruli to Aggravate Albuminuria in Diabetic Nephropathy. *Sci. Rep.* **6**, 20304; doi: 10.1038/srep20304 (2016).

## Supplementary Material

Supplementary Information
