# Supplementary material for: ROCK1 Induces Endothelial-to-Mesenchymal Transition in Glomeruli to Aggravate Albuminuria in Diabetic Nephropathy
Source: Sci Rep. 2016 Feb 4;6:20304. doi: 10.1038/srep20304 (PMC4740844; doi:10.1038/srep20304)
Supplement: Supplementary Information [file srep20304-s1.pdf]

ROCK1 Induces Endothelial-to-Mesenchymal Transition in Glomeruli to Aggravate Albuminuria in Diabetic Nephropathy

Hui Peng, Yuanqing Li, Cheng Wang, Jun Zhang, Yanru Chen, Wenfang Chen, Jin Cao, Yanlin Wang, Zhaoyong Hu*, Tanqi Lou*

Supplementary Information

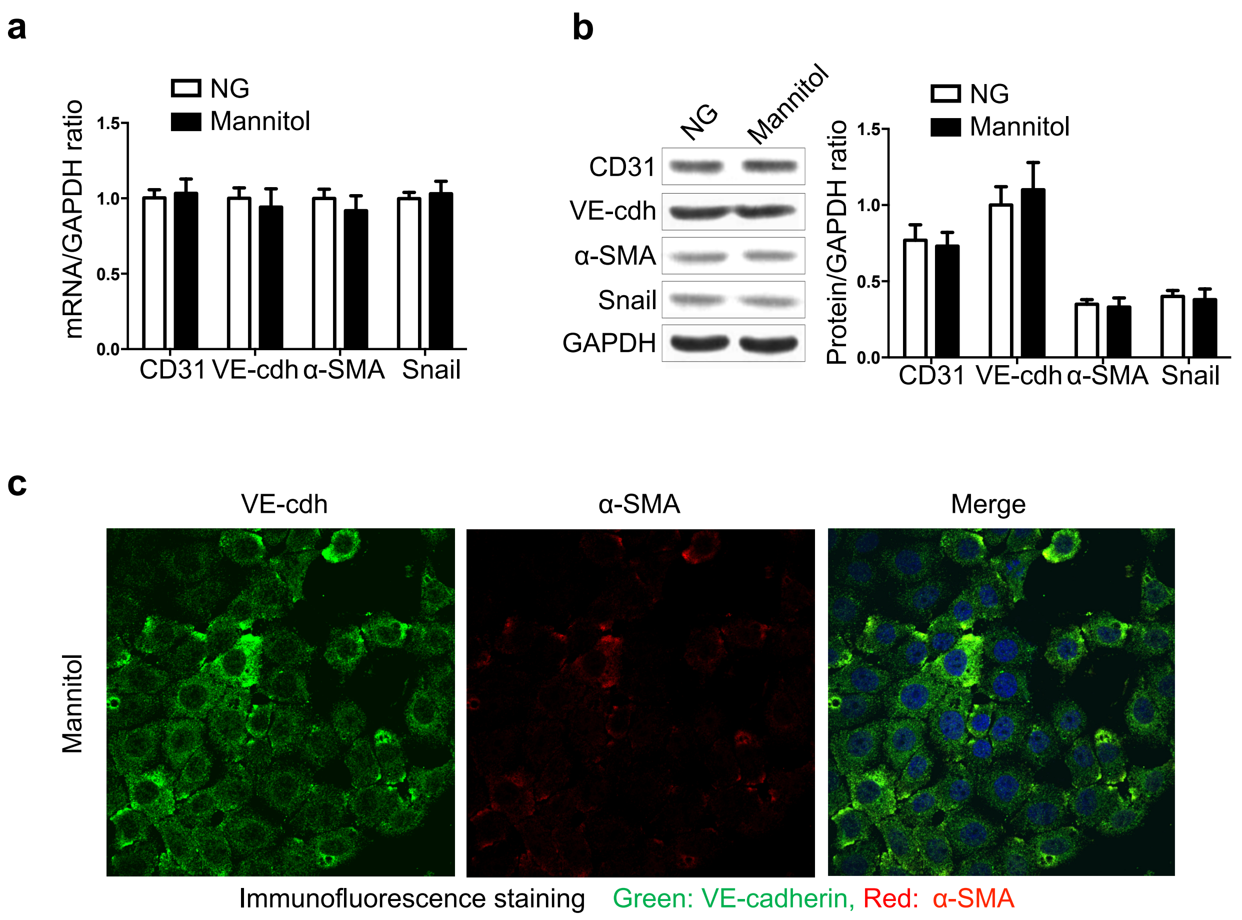

Supplementary Figure S1. Hyperosmolarity does not induce EndMT in cultured GEnCs. GEnCs were treated with 5.5 mM glucose or 5.5 mM glucose plus 24.5 mM mannitol for 5 days. **a**: mRNA levels of endothelial markers (VE-cadherin, CD31) and mesenchymal markers (α-SMA, Snail) were accessed using real-time PCR. **b**: protein levels of VE-cadherin, CD31, α-SMA and Snail were accessed with immunoblotting. **c**: Immunofluorescence double-staining of VE-cadherin (green) and α-SMA (red) in GEnCs incubated in glucose/mannitol mixture. NG: 5.5 mM glucose. Mannitol: 5.5 mM glucose + 24.5 mM mannitol.
